# Efficacy of power‐driven interdental cleaning tools: A systematic review and meta‐analysis

**DOI:** 10.1002/cre2.691

**Published:** 2022-12-23

**Authors:** Pia Edlund, Kristina Bertl, Nikolaos Pandis, Andreas Stavropoulos

**Affiliations:** ^1^ Department of Periodontology, Faculty of Odontology University of Malmö Malmö Sweden; ^2^ Division of Oral Surgery, University Clinic of Dentistry Medical University of Vienna Vienna Austria; ^3^ Department of Orthodontics and Dentofacial Orthopedics, School of Dental Medicine University of Bern Bern Switzerland

**Keywords:** bleeding, gingivitis, interdental cleaning device, meta‐analysis, plaque, systematic review

## Abstract

**Objectives:**

To summarize the available evidence on the efficacy of power‐driven interdental cleaning tools (PDICTs) as an adjunct to tooth brushing compared to tooth brushing alone or tooth brushing combined with any other non‐PDICT in terms of interproximal plaque and gingival bleeding reduction in gingivitis patients.

**Material and Methods:**

A systematic literature search was performed in three databases until March 20, 2022 with the following main eligibility criteria: (1) randomized controlled clinical trials (RCTs) with (2) at least 28 days of follow‐up in (3) gingivitis patients. Interproximal plaque and bleeding values were defined as the primary outcome variables and used for pair‐wise meta‐analyses.

**Results:**

Sixteen RCTs were identified including data from 1258 participants at the final evaluation. Eight studies each investigated the effect of either a liquid‐based or mechanical PDICT; one of these studies tested additionally a combined liquid‐based and mechanical PDICT. Tooth brushing combined with a liquid‐based PDICT compared to tooth brushing alone did not result in better interproximal plaque values but in significantly lower interproximal bleeding values. Tooth brushing combined with either a liquid‐based PDICT or with a mechanical PDICT compared to tooth brushing and flossing achieved comparable interproximal plaque and bleeding values. The majority of studies reporting on patient compliance/preference favored the use of a PDICT, and except for a single study, which was reporting soft tissue trauma in two subjects from improper use of a mechanical PDICT, none of the studies reported adverse events.

**Conclusions:**

Daily use of PDICT as an adjunct to tooth brushing significantly reduces interproximal bleeding. This effect appears comparable to that of flossing, while PDICT may achieve higher patient acceptance/compliance.

## 1 BACKGROUND

Tooth brushing is the standard method for removing dental plaque (Addy et al., 1986; Mak & Day, 2011; Richardson et al., 1977). However, it has only a marginal effect on the interdental spaces, which are often narrow and difficult to clean and therefore prone to develop caries, gingivitis, and periodontitis (Galgut, 1991). There are numerous interdental cleaning tools (ICTs) in the market to be used as an adjunct to tooth brushing, with the aim to improve the patient's oral health care and reduce the risk for disease. In theory, the choice of the device should be site‐specific, depending on interdental space accessibility, which in turn is defined by local anatomical (e.g., tooth position/malposition) and iatrogenic (e.g., prosthetic restorations, orthodontic appliances, etc.) factors, that is, not all ICT suit all patients and situations. Nevertheless, flossing remains a frequently recommended standard by dental professionals, especially for children and adolescents (Särner et al., 2010), although studies have indeed shown better plaque removal with interdental brushes, followed by toothpicks (Sälzer et al., 2015; Schmid et al., 1976; Yost et al., 2006).

In this context, another important aspect is patient compliance in terms of using an ICT systematically and consistently, which is often difficult to achieve (Smith et al., 2019). For example, it is reported that only about 1/3rd of patients floss once a day, irrespective of age (W. P. Lang et al., 1995; Srinivasan et al., 2019; Winterfeld et al., 2015), while in a survey among >2000 US adults, it was disclosed that about 1/4th of the patients tend to lie to their dentists about their flossing habits. Additionally, 36% would prefer doing other unpleasant activities instead of daily flossing (e.g., cleaning the toilet, working on the taxes, or washing a sink full of dirty dishes) (American Academy of Periodontology, 2019). Recent studies have shown that power‐driven ICTs (PDICTs), either liquid‐based, mechanical, or a combination thereof, potentially achieve a higher patient preference compared to other ICT (e.g., flossing) (Bertl, Edlund Johansson et al., 2021; Lyle, 2012; Sharma et al., 2008; Shibly et al., 2001). Liquid‐based PDICT have been introduced in the 1960–1970s, with the first oral irrigator aiming to clean the interdental space with a water jet (Lobene, 1969). Modern power‐driven oral irrigators (e.g., WaterPik, Water Pik, Inc.) and air/liquid flossers (e.g., Sonicare AirFloss) use either a stream of liquid or a stream of air with liquid microdroplets, with either low or high pressure, to remove plaque. Both types can be used with water only or with antibacterial and/or anti‐inflammatory mouth rinses, such as chlorhexidine (Flemmig et al., 1990; N. P. Lang & Raber, 1981). Mechanical PDICT, on the other hand, are basically electric vibrating or rotating interdental brushes, toothpicks, and flossers.

In general, PDICTs have not achieved wide acceptance within the dental community, which may partly be due to the lack of any comprehensive systematic appraisal of the literature about their clinical efficacy (i.e., plaque removal and prevention of gingivitis). Thus, the aim of the present systematic review was to answer the following focused question according to the Population, Intervention, Comparison, Outcomes, Study Design criteria (Miller & Forrest, 2001): “In gingivitis patients (P), what is the efficacy of any type of PDICT (e.g., liquid‐based, mechanical, and/or combinations thereof) (I) as an adjunct to tooth brushing compared to tooth brushing alone (C1) or compared to tooth brushing combined with any other non‐PDICT (NPDICT) (C2) in terms of plaque (O1) and gingival bleeding levels (O2)?”

## 2 MATERIALS AND METHODS

### 2.1 Protocol and eligibility criteria

The present systematic review followed the criteria of the Preferred Reporting Items for Systematic Reviews and Meta‐analyses (Supporting Information: Appendix 1) (Page et al., 2021). The following inclusion criteria were applied: (1) articles written in English and published in peer‐reviewed journals; (2) randomized controlled clinical trials (RCTs) with (3) at least 28 days of follow‐up and (4) with the purpose to investigate the efficacy of PDICT as an adjunct to any type of tooth brushing (5) compared to tooth brushing alone or compared to tooth brushing combined with any NPDICT (6) in gingivitis patients (7) in terms of plaque and gingival bleeding reduction. Studies including patients with periodontitis, undergoing orthodontic treatment, or with psychological or physical disability, as well as studies focusing on implants, or in vitro, laboratory, and preclinical studies were excluded.

### 2.2 Information sources and literature search

The search process included originally two sources (MEDLINE/PubMed and the Cochrane central register of controlled trials [CENTRAL]), which were screened for articles from 1980 up to and including April 2020. The search was updated before submission for the period April 1, 2020 to March 20, 2022, and during the revision process for a third source covering the same time period (Embase). Details on the search term are presented in Supporting Information: Appendix 2. A manual search from relevant literature and previous review articles was also performed. Finally, a forward search via Science Citation Index with the included papers was added.

### 2.3 Data collection and extraction

Based on the above‐listed eligibility criteria, the titles, abstracts, and finally full‐texts were screened for relevance by two authors (P. E. J. and K. B.); if no abstract was available, the article was read in full‐text. In case of ambiguity, consensus through discussion was achieved together with a third author (A. S.). The following clinical parameters were extracted by two authors (P. E. J. and K. B.) from the included studies at baseline and at final evaluation for statistical analysis: plaque, bleeding, and gingival indices at interproximal sites only and as mean of all measured sites. Additionally, the following study details were extracted and summarized in tables, if reported: study design, number of participants, type of ICT investigated, type of intervention and control treatment, duration of the trial, loss to follow‐up, patient preferences, adverse events related to the ICT, randomization process, blinding process, and participants' demographics, and overall and oral health status.

### 2.4 Risk of bias assessment

All included articles were assessed for risk of bias using the RoB2 tool from Cochrane (Sterne et al., 2019). The following domains were evaluated at “low,” “high,” or “unclear” risk of bias: risk of bias arising from (1) the randomization process, (2) deviations from the intended interventions (*effect of adhering to intervention*), (3) missing outcome data, (4) measurement of the outcome, and (5) selection of the reported results. As the specific research question did not allow a proper blinding process, the effect of adhering instead of assignment to intervention was judged in the second domain. It was considered as unclear if no follow‐up on adherence to the study protocol was reported and low if it was reported and not rising any concerns. The overall risk of bias for an individual study was judged as follows: low, if all criteria were evaluated to be of low risk; high, if at least one criterion was evaluated to be of high risk; and unclear, if at least one criterion was evaluated to be of unclear risk but no criterion of high risk.

### 2.5 Synthesis of results

The following six comparisons were considered for grouping the studies:

(1) Comparison 1: Brushing versus brushing + liquid‐based PDICT.
(2) Comparison 2: Brushing + flossing versus brushing + liquid‐based PDICT.
(3) Comparison 3: Brushing versus brushing + mechanical PDICT.
(4) Comparison 4: Brushing + flossing versus brushing + mechanical PDICT.
(5) Comparison 5: Brushing versus brushing + combined liquid‐based and mechanical PDICT.
(6) Comparison 6: Brushing + flossing versus brushing + combined liquid‐based and mechanical PDICT.

Two primary outcome variables (i.e., interproximal plaque values and interproximal bleeding values) and several secondary outcome variables (i.e., interproximal gingival index, and plaque, bleeding, and gingival indices as mean of all measured sites) were defined. If necessary, outcome variable values were calculated, for example, by calculating the mean of the buccal and lingual interproximal values to represent the overall interproximal value of the specific side.

### 2.6 Statistical analysis

For the above‐listed outcome parameters and comparisons, random‐effects pair‐wise meta‐analyses using the inverse variance method with a comparable follow‐up period (i.e., either approximately 30 days or ≥90 days) were implemented. Restricted maximum likelihood [REML] to calculate heterogeneity (*τ* ^2^) was used and the Knapp–Hartung standard error adjustment to account for the small number of studies. Standardized mean differences (SMD: Hedges's *g*) were used to compensate for the different indices recorded in the original studies. In case of at least three studies, the 95% prediction interval was estimated and displayed in the forest plots. Statistical analysis was performed using statistical software (STATA/IC 17.0 for Mac, StataCorp LLC). Finally, the GRADEpro GDT (Guideline Development Tool, McMaster University and Evidence Prime, 2022) software was used to grade the quality of evidence of the results (Chen et al., 2015).

## 3 RESULTS

### 3.1 Study selection

**Figure 1:**
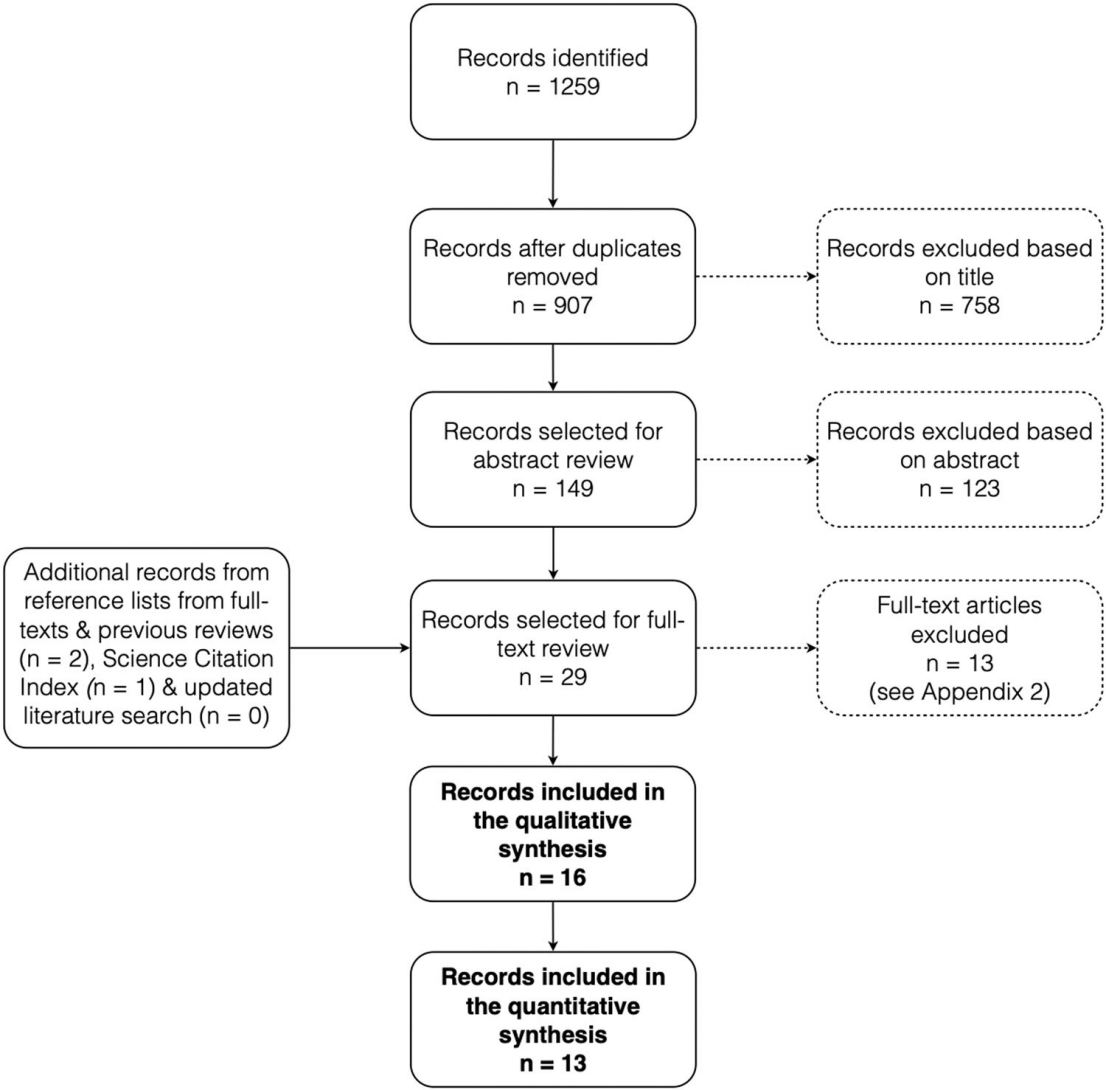
Flowchart of the inclusion process of studies for the systematic review

A flowchart of the literature search is presented in Figure 1. In total, 1259 titles were identified, and after screening, 29 publications were read in full text and assessed for eligibility; 13 publications were excluded for various reasons (Supporting Information: Appendix 3). The literature search covering the period April 1, 2020 until March 20, 2022 resulted in additional 68 references, of which one was relevant but did not fulfill the eligibility criteria (Ramseier et al., 2021), that is, the two groups used different toothbrushes. The literature search in the third database performed during the revision process did not identify any relevant study for inclusion. Hence, 16 RCTs were included in this review (Anderson et al., 1995; Barnes et al., 2005; Cronin & Dembling, 1996; Cronin et al., 1997, 2005; Frascella et al., 2000; Gordon et al., 1996; Goyal et al., 2012, 2018; Hague & Carr, 2007; Isaacs et al., 1999; Lyle et al., 2020; Rosema et al., 2011; Shibly et al., 2001; Stauff et al., 2018; Walsh et al., 1989) in the qualitative synthesis, while 13 studies could be included in the quantitative synthesis (Anderson et al., 1995; Barnes et al., 2005; Cronin et al., 1997, 2005; Frascella et al., 2000; Gordon et al., 1996; Goyal et al., 2012, 2018; Hague & Carr, 2007; Lyle et al., 2020; Rosema et al., 2011; Shibly et al., 2001; Stauff et al., 2018). During the full‐text review, both reviewers (P. E. J. and K. B.) agreed 100% on the RCT to be included (Cohen's *κ* of 1).

### 3.2 Study characteristics

An overview of the study design, study population, type of intervention and interdental tools used, and outcome measures applied is summarized below and provided in Table 1.

**Table 1:** Overview of the included studies

		Study population				
		Sample size (*n*)				
		Male/female (*n*)	Follow‐up period (days)			
Study Author (year)	Study design	Age (range, mean)	Loss to follow‐up (*n*)	Control group	Test group	Outcome measures
Comparison 1: Brushing versus brushing + liquid‐based PDICT						
Walsh et al. (1989)	RCT parallel group Single blind Ramfjord teeth^a^	108 NR 18–65, NR	180 0	MTB PTB	MTB + OI (Broxojet, Advanced Response Corporation) PTB + OI (Broxojet)	Plaque (Silness and Löe) Gingivitis (Löe and Silness) Bleeding (Löe and Silness)
Frascella et al. (2000)	RCT parallel group Single blind Full mouth	56 22/42 18–65, 39.5	56 8	MTB	MTB + OI (Oral‐B Oxyjet, The Procter & Gamble Company)	Plaque (TQHPI) Gingivitis (MGI) Bleeding (ABI)
Goyal et al. (2012)	RCT parallel group Single blind Full mouth	140 44/96 25–65, 44.4	30 1	PTB	PTB + OI (WaterPik complete care, Water Pik, Inc.)	Plaque (RMNPI) Gingivitis (MGI)
Goyal et al. (2018)	RCT parallel group Single blind Full mouth	72 16/56 25–70, 48.4	28 0	MTB	MTB + OI (WaterPik Water Flosser, Water Pik, Inc.)	Plaque (RMNPI) Gingivitis (MGI) Bleeding (BoP)
Lyle et al. (2020)	RCT parallel group Single blind Full mouth	70 25/45 18–70, 43.1	30 0	PTB	PTB + OI (WaterPik Aquarious, Water Pik, Inc.)	Plaque (RMNPI) Gingivitis (MGI) Bleeding (BoP)
Comparison 2: Brushing + flossing versus brushing + liquid‐based PDICT						
Barnes et al. (2005)	RCT parallel group Single blind Full mouth	108 NR 19–70, NR	28 10	MTB + FL	MTB + OI (WaterPik dental water jet, Water Pik, Inc) PTB + OI (WaterPik dental water jet)	Plaque (proximal/marginal PI) Gingivitis (Löe and Silness) Bleeding (Carter and Barnes)
Rosema et al. (2011)^b^	RCT parallel group Single blind Full mouth	108 30/75 >18, 21.8	30 4	MTB + FL	MTB + OI standard (WaterPik with standard tip)	Plaque (TQHPI) Gingivitis (BOMP)
Stauff et al. (2018)	RCT parallel group Single blind Premolars	60 25/35 18–46, 27.6	28 1	MTB + FL	MTB + OI (Philips Sonicare AirFloss Ultra)	Plaque (MPPI) Bleeding (PBI)
Comparison 3: Brushing versus brushing + mechanical PDICT						
Hague and Carr (2007)^c^	RCT parallel group and cross over Single blind Full mouth	102 34/68 NR, 23.3	30 13	MTB	MTB + PFL (William Getgey Co. Ultra Flosser)	Plaque (TQHPI) Gingivitis (Löe and Silness)
Comparison 4: Brushing + flossing versus brushing + mechanical PDICT						
Anderson et al. (1995)	RCT parallel group Single blind Full mouth	57 NR 18–65, NR	30 3	MTB + FL	MTB + PFL (Sky Vision Inc., Power flosser)	Plaque (proximal/marginal PI) Bleeding (modified PBI)
Cronin and Dembling (1996)	RCT parallel group Single blind Full mouth	48 17/31 18–65, 36	42 6	MTB + FL	MTB + PIC (Oral‐B Interclean, The Procter & Gamble Company)	Plaque (proximal/marginal PI) Gingivitis (MGI) Bleeding (ABI)
Gordon et al. (1996)	RCT parallel group Single blind Full mouth	52 NR 24–45, NR	30 8	MTB + FL	MTB + PIC (Oral‐B Interclean)	Plaque (proximal/marginal PI) Gingivitis (MGI) Bleeding (modified PBI)
Cronin et al. (1997)	RCT parallel group Single blind Full mouth	60 16/43 18–65, 36	28 1	MTB + FL	MTB + PIC (Oral‐B Interclean with Flexi‐Tip)	Plaque (TQHPI) Gingivitis (Löe and Silness) Bleeding (Löe and Silness)
Isaacs et al. (1999)	RCT parallel group Single blind Full mouth	147 NR 18–NR, NR	90 23	MTB + FL	MTB + PIC (Oral‐B Interclean)	Gingivitis (MGI) Bleeding (ABI)
Shibly et al. (2001)	RCT parallel group Single blind Full mouth	70 20/50 NR, 37.6	30 0	MTB + FL	MTB + PFL (WaterPik Power Flosser, Water Pik, Inc.)	Plaque (NR) Gingivitis (modified GI) Bleeding (Eastman)
Cronin et al. (2005)	RCT parallel group Single blind Full mouth	84 23/55 18–70, 36	30 6	MTB + FL	MTB + PTP (Oral‐B Hummingbird powerpick, The Procter & Gamble Company) MTB + PFL (Oral‐B Hummingbird powerfloss,The Procter & Gamble Company)	Plaque (proximal/marginal PI) Gingivitis (Löe and Silness) Bleeding (Löe and Silness)
Hague and Carr (2007)^c^	RCT parallel group and cross over Single blind Full mouth	102 34/68 NR, 23.3	30 13	MTB + FL	MTB + PFL (William Getgey Co. Ultra Flosser)	Plaque (TQHPI) Gingivitis (Löe and Silness)
Comparison 6: Brushing + flossing versus brushing + combined liquid‐based and mechanical PDICT						
Rosema et al. (2011)^b^	RCT parallel group Single blind Full mouth	108 30/75 >18, 21.8	30 4	MTB + FL	MTB + OI prototype (WaterPik with prototype jet tip)	Plaque (TQHPI) Gingivitis (BOMP)

Abbreviations: ABI, angular bleeding index; BOMP, bleeding on marginal probing; BoP, bleeding on probing; FL, flossing; GI, gingival index; MGI, modified gingival index; MPPI, modified proximal plaque index; MTB, manual toothbrush; NR, not reported; OI, oral irrigator; PBI, papilla bleeding index; PDICT, power‐driven interdental cleaning tool; PFL, powered flosser; PI, plaque index; PIC, powered interdental cleaner; PTB, powered toothbrush; PTP, powered toothpick; RCT, randomized controlled clinical trial; RMNPI, Rustogi modification of the Navy plaque index; TQHPI, Turesky modification of the Quigley and Hein plaque index.

^a^ The Ramfjord teeth include tooth numbers 16, 21, 24, 36, 41, and 44.

^b^ Rosema et al. (2011) was contributing to Comparisons 2 and 6.

^c^ Hague and Carr (2007) was contributing to Comparisons 3 and 4.

#### 3.2.1 Study design

All studies were single‐blinded and arranged in parallel groups, with a single study (Hague & Carr, 2007) applying a cross‐over design. Further, all studies assessed full‐mouth values, except for two studies, that either used the first premolars as study teeth (Stauff et al., 2018) or the Ramfjord teeth, that is, tooth numbers 16, 21, 24, 36, 41, and 44 (Walsh et al., 1989). The follow‐up period ranged from 28 to 180 days, with 12 studies having a follow‐up of 28–30 days, one study reporting data after 28 and 56 days, and three studies with a follow‐up of 42, 90, or 180 days, respectively.

#### 3.2.2 Study population

The sample size of the individual studies ranged from 48 to 147 patients. Herein, the data of 1258 participants are summarized (1342 participants in total at baseline, with 84 lost during follow‐up). No study included patients <18 years old and the mean age—if reported—varied from 22 to 48 years.

#### 3.2.3 Interventions

Tooth brushing was performed in two studies (Goyal et al., 2012; Lyle et al., 2020) with a powered toothbrush, while in 13 studies with a manual toothbrush; one study (Walsh et al., 1989) had four groups, that is, two groups used a manual and two a powered toothbrush. In the control groups of five studies (Frascella et al., 2000; Goyal et al., 2012, 2018; Lyle et al., 2020; Walsh et al., 1989) brushing alone was performed (i.e., no interdental cleaning), while in 10 studies the participants of the control group used brushing and flossing for interdental cleaning; one study (Hague & Carr, 2007) had two control groups, that is, brushing alone and brushing with flossing. Eight studies investigated the efficacy of a liquid‐based PDICT as an adjunct to tooth brushing, of which five studies used a WaterPik device (different models) and one study each Broxojet, Advanced Response Corporation, Oral‐B Oxyjet, The Procter & Gamble Company, and Philips Sonicare AirFloss Ultra, Royal Philips N.V. One of these studies tested additionally a prototype, which was classified as a combined liquid‐based and mechanical PDICT. Further, eight studies investigated the efficacy of a mechanical PDICT as an adjunct to tooth brushing; four of these mechanical PDICT devices were classified as powered flossers, one as a powered toothpick, and two different types of powered interdental cleaners.

#### 3.2.4 Outcome measures

Fifteen studies reported on various plaque indices, 14 studies on various gingival indices, and 13 studies on various bleeding indices. Primarily mean interproximal and/or full mouth scores were reported, while some studies also reported separately buccal and palatal/lingual scores.

Six studies (Gordon et al., 1996; Hague & Carr, 2007; Lyle et al., 2020; Shibly et al., 2001; Stauff et al., 2018; Walsh et al., 1989) reported on the compliance and/or patient preference (Table 2). In three studies (Lyle et al., 2020; Stauff et al., 2018; Walsh et al., 1989), more participants of the groups using a PDICT indicated continuing to use it. Two studies (Gordon et al., 1996; Shibly et al., 2001) indicated a clearly higher preference for the mechanical PDICT compared to flossing, and in one study (Stauff et al., 2018), the participants rated the liquid‐based PDICT more comfortable, but less effective compared to flossing. All studies reported on the occurrence of adverse events related to the ICT, and in all but one, no adverse events occurred; a single study (Hague & Carr, 2007) reported soft tissue trauma from improper use of the mechanical PDICT, in two subjects.

**Table 2:** Summary of patient preferences and adverse events related to the ICT

Study Author (year)	Control group	Test group	Patient preference	Adverse events related to the ICT
Comparison 1: Brushing versus brushing + liquid‐based PDICT				
Walsh et al. (1989)	MTB	MTB + OI (Broxojet, Advanced Response Corporation)	Compliance in all four groups was excellent; 100% of PTB + OI, 73% of PTB, 86% of MBI + OI, and 90% of MBT were satisfied and planned to continue using it.	No adverse events
	PTB	PTB + OI (Broxojet)		
Frascella et al. (2000)	MTB	MTB + OI (Oral‐B Oxyjet, The Procter & Gamble Company)	NR	No adverse events
Goyal et al. (2012)	PTB	PTB + OI (WaterPik complete care, Water Pik, Inc.)	NR	No adverse events
Goyal et al. (2018)	MTB	MTB + OI (WaterPik Water Flosser, Water Pik, Inc.)	NR	No adverse events
Lyle et al. (2020)	PTB	PTB + OI (WaterPik Aquarious, Water Pik, Inc.)	Subjects in both groups felt the device was easy to use, the instructions were clear, their mouth felt fresh and clean, and they would recommend the product to family and friends. Significantly more participants of the test group would continue to use the products after the study (91% vs. 60%).	No adverse events
Comparison 2: Brushing + flossing versus brushing + liquid‐based PDICT				
Barnes et al. (2005)	MTB + FL	MTB + OI (WaterPik dental water jet, Water Pik, Inc) PTB + OI (WaterPik dental water jet)	NR	No adverse events
Rosema et al. (2011)^a^	MTB + FL	MTB + OI standard (WaterPik with standard tip)	NR	No adverse events
Stauff et al. (2018)	MTB + FL	MTB + OI (Philips Sonicare AirFloss Ultra)	The microdroplet device was rated more comfortable (83% vs. 50%), and fewer signs of inflammation (blood) after brushing were reported by participants. The self‐reported effectiveness was superior in the dental floss group (79% vs. 100%). More participants using the microdroplet device said they would continue daily use (84% vs. 75%). Adherence to the daily interdental cleaning routine was slightly better in the microdroplet device group (85% vs. 79%).	No adverse events
Comparison 3: Brushing versus brushing + mechanical PDICT				
Hague and Carr (2007)^b^	MTB	MTB + PFL (William Getgey Co. Ultra Flosser)	The rate of compliance was comparable. Preference not reported.	Soft tissue trauma from improper use of the automated flossing device in two subjects.
Comparison 4: Brushing + flossing versus brushing + mechanical PDICT				
Anderson et al. (1995)	MTB + FL	MTB + PFL (Sky Vision Inc. Power flosser)	NR	No adverse events
Cronin and Dembling (1996)	MTB + FL	MTB + PIC (Oral‐B Interclean, The Procter & Gamble Company)	NR	No adverse events, but two subjects of the test group expressed some concern about filament breakage, but this was considered as of no clinical significance.
Gordon et al. (1996)	MTB + FL	MTB + PIC (Oral‐B Interclean)	69% preferred the PDICT over flossing, 25% preferred flossing over PDICT, and 6% had no preference. The reasons for preferring PDICT focused on ease of use with ready access to the posterior teeth.	No adverse events
Cronin et al. (1997)	MTB + FL	MTB + PIC (Oral‐B Interclean with Flexi‐Tip)	NR	No adverse events
Isaacs et al. (1999)	MTB + FL	MTB + PIC (Oral‐B Interclean)	NR	No adverse events.
Shibly et al. (2001)	MTB + FL	MTB + PFL (WaterPik Power Flosser)	No significant difference in comfort index score, but over 80% of initial manual flossers preferred the PDICT, and over 95% of the initial PDICT preferred the PDICT.	No adverse events.
Cronin et al. (2005)	MTB + FL	MTB + PTP (Oral‐B Hummingbird powerpick, The Procter & Gamble Company) MTB + PFL (Oral‐B Hummingbird powerfloss, The Procter & Gamble Company)	NR	No adverse events
Hague and Carr (2007)^b^	MTB + FL	MTB + PFL (William Getgey Co. Ultra Flosser)	The rate of compliance was comparable. Preference not reported.	Soft tissue trauma from improper use of the automated flossing device in two subjects.
Comparison 6: Brushing + flossing versus brushing + combined liquid‐based and mechanical PDICT				
Rosema et al. (2011)^a^	MTB + FL	MTB + OI prototype (WaterPik with prototype jet tip)	NR	No adverse events

Abbreviations: FL, flossing; ICT, interdental cleaning tool; MTB, manual toothbrush; NR, not reported; OI, oral irrigator; PDICT, power‐driven interdental cleaning tool; PFL, powered flosser; PIC, powered interdental cleaner; PTB, powered toothbrush; PTP, powered toothpick.

^a^ Rosema et al. (2011) was contributing to Comparisons 2 and 6.

^b^ Hague and Carr (2007) was contributing to Comparisons 3 and 4.

### 3.3 Risk of bias assessment

Except for a single study (Goyal et al., 2018), which had a low risk of bias, all studies presented overall with an unclear risk of bias. Mostly Domain 1 (randomization process) and Domain 5 (selection of the reported results) presented some concerns, either due to lack of information on the randomization process or due to lack of an a priori published study protocol allowing to compare the intended and finally performed the statistical analysis. For an overview see Supporting Information: Appendix 4.

### 3.4 Synthesis of results

All studies reporting data at approximately 30 days were pooled for meta‐analyses. Meta‐analyses were possible for at least one of the outcome variables regarding Comparisons 1 (i.e., brushing vs. brushing + liquid‐based PDICT), 2 (i.e., brushing + flossing vs. brushing  + liquid‐based PDICT), and 4 (i.e., brushing + flossing vs. brushing + mechanical PDICT). No meta‐analyses were possible for Comparison 3 (i.e., brushing vs. brushing + mechanical PDICT), 5 (i.e., brushing vs. brushing + combined liquid‐based and mechanical PDICT), and 6 (i.e., brushing + flossing vs. brushing + combined liquid‐based and mechanical PDICT), because either no (Comparison 5) or only one study (Comparisons 3 [Hague & Carr, 2007] and 6 [Rosema et al., 2011]) was identified. No meta‐analyses were feasible for longer follow‐up times due to limited number of studies/groups.

A summary of the analyses on the two primary outcome variables is given below, while the secondary outcome variables are summarized in Supporting Information: Appendix 5.

#### 3.4.1 Comparison 1: Brushing versus brushing + liquid‐based PDICT

**Figure 2:**
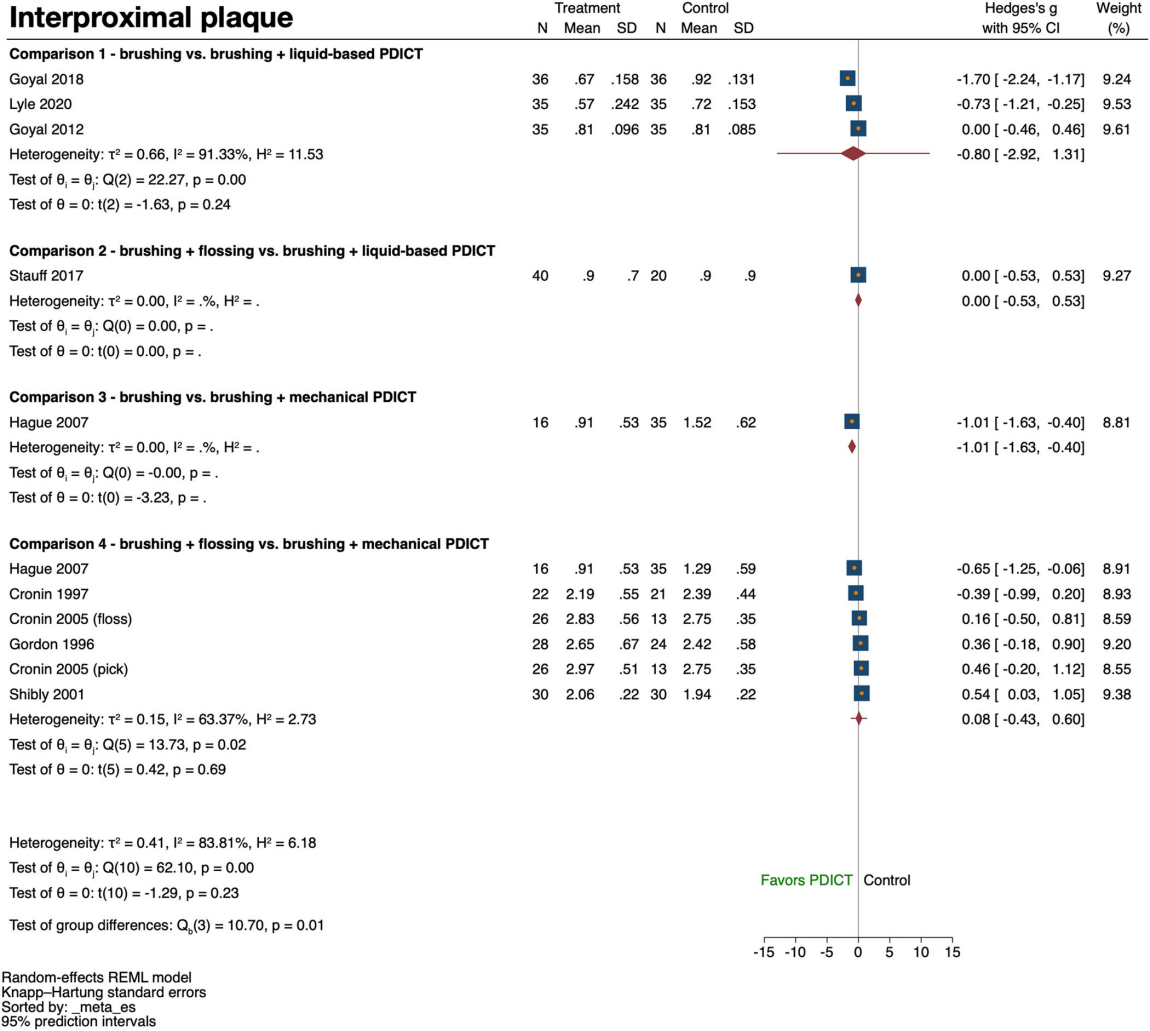
Forest plot for interproximal plaque

**Figure 3:**
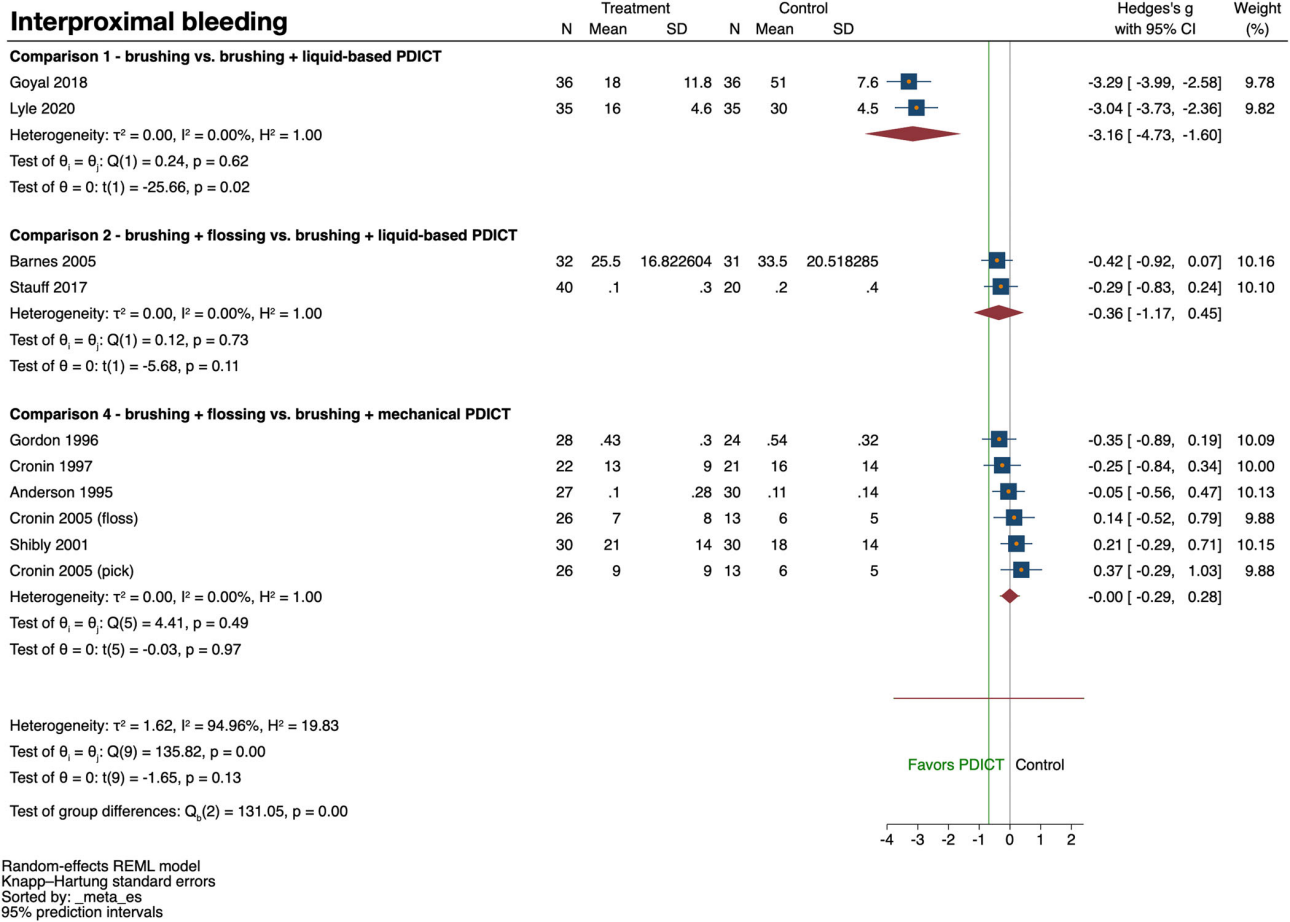
Forest plot for interproximal bleeding

Three studies (Goyal et al., 2012, 2018; Lyle et al., 2020) assessed interproximal plaque. Two studies reported a positive effect of the adjunct use of a liquid‐based PDICT (Goyal et al., 2018; Lyle et al., 2020), while the third study lacked any relevant difference between the groups. A high heterogeneity was detected among the studies (*I* ^2^= 91.3%, *p* < .01) and meta‐analysis failed to show a significant effect of the adjunct use of a liquid‐based PDICT (SMD: −0.80; 95% confidence interval [CI]: −2.92, 1.31; *p* = .24) (Figure 2). The quality of evidence was judged as very low (Supporting Information: Appendix 6a). Two studies (Goyal et al., 2018; Lyle et al., 2020) assessed interproximal bleeding. Both studies presented a positive effect of the adjunct use of PDICT without significant heterogeneity among the studies (*I* ^2^ = 0%, *p* = .62). The meta‐analysis showed an overall significant positive effect favoring the adjunct use of a liquid‐based PDICT (SMD: −3.16; 95% CI: −4.73, −1.60; *p* = .02) (Figure 3). The quality of evidence was judged as moderate (Supporting Information: Appendix 6a).

#### 3.4.2 Comparison 2: Brushing + flossing versus brushing + liquid‐based PDICT

Two studies (Barnes et al., 2005; Stauff et al., 2018) assessed interproximal bleeding. Although both studies presented a slightly superior effect with the use of a liquid‐based PDICT compared to flossing without significant heterogeneity among the studies (*I* ^2^ = 0%, *p* = .73), meta‐analysis failed to show a positive effect favoring the use of a liquid‐based PDICT (SMD: −0.36; 95% CI: −1.17, 0.45; *p* = .11) (Figure 3). The quality of evidence was judged as low (Supporting Information: Appendix 6b).

#### 3.4.3 Comparison 4: Brushing + flossing versus brushing + mechanical PDICT

Five studies (Cronin et al., 1997, 2005; Gordon et al., 1996; Hague & Carr, 2007; Shibly et al., 2001) contributing with six comparisons assessed interproximal plaque. Only two studies favored slightly the use of a PDICT and there was significant heterogeneity among the studies (*I* ^2^ = 63.4%, *p* = .02). Meta‐analysis did not show a significant difference between the two interventions (SMD: 0.08; 95% CI: −0.43, 0.60; *p* = .69) (Figure 2). The quality of evidence was judged as low (Supporting Information: Appendix 6c). Five studies (Anderson et al., 1995; Cronin et al., 1997, 2005; Gordon et al., 1996; Shibly et al., 2001) contributing with six comparisons assessed interproximal bleeding, presenting inconsistent results in terms of a better outcome with the use of a PDICT. Meta‐analysis yielded no significant difference between the two interventions (SMD: 0.00; 95% CI: −0.29, 0.28; *p* = .97) with no significant heterogeneity among the studies (*I* ^2^ = 0%, *p* = .49) (Figure 3). The quality of evidence was judged as low (Supporting Information: Appendix 6c).

## 4 DISCUSSION

The present study represents a comprehensive systematic appraisal of the literature on the clinical efficacy of PDICT in terms of plaque and bleeding in gingivitis patients. The results indicated that daily use of a liquid‐based PDICT as adjunct to brushing significantly reduces gingival inflammation, as captured by interproximal and full‐mouth bleeding indices; however, this seems not to be due to better plaque control, as captured by plaque indices. Furthermore, it appears that the clinical efficacy in terms of plaque and bleeding control of either liquid‐based or mechanical PDICT is well comparable to flossing as an adjunct to brushing.

These findings are in general in line with those of previous reviews (Husseini et al., 2008; Ng & Lim, 2019; Worthington et al., 2019) showing that compared to brushing alone, the use of a liquid‐based PDICT significantly improves gingival health, while in regards with plaque control, inconsistent results are obtained. The finding that the use of an ICT as an adjunct to brushing results in improved gingival health comes without surprise; it is well established that interdental cleaning for plaque removal is pivotal to prevent oral diseases such as caries, gingivitis, and periodontitis in susceptible individuals (Chapple et al., 2015; Sälzer et al., 2015, 2020). The finding, however, that the positive effect of liquid‐based PDICT on interproximal bleeding scores is not associated with reduced amounts of interproximal plaque was indeed unexpected. It has been previously discussed that this observation may be due to an impact of PDICT on biofilm composition, thickness and/or maturation, and/or a stimulated immune response (for an overview, see Husseini et al., 2008); however, a proper, purpose‐designed study is missing.

In this context, although PDICT did not show any clinical benefit compared to flossing, they might have advantages in terms of patient compliance/preference. In all six studies included in this review and reporting on patient compliance and/or preferences, PDICT scored most often clearly better. A similar higher preference for and/or more often the wish to continue to use a liquid‐based PDICT has been reported in a recent study from our group, not included in the present review (Bertl, Edlund Johansson et al., 2021). As already mentioned, patient compliance in terms of using an ICT systematically and consistently is a matter of concern (Smith et al., 2019) and only a small fraction of patients floss daily (W. P. Lang et al., 1995; Srinivasan et al., 2019; Winterfeld et al., 2015), while a considerable number tends to lie to their dentists about their flossing habits (Periodontology, 2019). It may thus seem reasonable to assume that the use of PDICT may be beneficial in the long term in controlling/preventing gingivitis/periodontitis, by facilitating better patient compliance; however, this has yet to be assessed in properly designed RCT with long‐term follow‐up. Nevertheless, in the only two studies (Cronin & Dembling, 1996; Isaacs et al., 1999) with a longer follow‐up (i.e., 42–90 days) identified herein, mechanical PDICT was equally effective in flossing. In this context, based on a recent network meta‐analysis (Kotsakis et al., 2018) ranking the efficacy of the various ICT, liquid‐based PDICTs were judged as the second most effective adjunct to brushing to reduce both bleeding and gingival indices. Toothpicks with intensive oral hygiene instructions ranked first for bleeding, and interdental brushes ranked first for gingival indices and plaque reduction.

In perspective, any ICT should be safe to use. In general, all studies included herein reported that PDICTs are indeed safe to use. Nevertheless, recent reports from our group have indicated that liquid‐based PDICTs are unavoidably colonized by oral bacteria, which can be transmitted via the water jet (Bertl, Edlund Johansson, Bruckmann et al., 2021; Bertl et al., 2022). Contamination of a specific liquid‐based PDICT with *Streptococcus mutans* was found in >95% of the samples, while periodontal pathogens were detected in 19%–56% of the samples (Bertl et al., 2022). Based on these reports, the commonly suggested use of the same device within families, with only exchanging the device tip among family members, should be reconsidered. Specifically, it should be suggested that each family member has their own device. Whether similar concerns, in terms of bacterial colonization, apply also to other liquid‐based PDICT remains to be assessed.

The literature available on PDICT has of course some limitations, impacting the generalizability of the results of the present review. Specifically, conclusions are limited to short term (i.e., about 1‐month follow‐up), as there were only two studies (Frascella et al., 2000; Walsh et al., 1989) with a long‐term follow‐up (i.e., 56–180 days). These studies did not show any benefit of adjunct use of a liquid‐based PDICT compared to brushing alone; however, both studies did not present specifically interproximal plaque values, thus a strong conclusion on the impact of PDICT should not be drawn. Furthermore, only three specific comparisons (i.e., brushing vs. brushing + liquid‐based PDICT, brushing + flossing vs. brushing + liquid‐based PDICT, and brushing + flossing vs. brushing + mechanical PDICT) could be assessed herein. Interestingly, no studies have compared PDICT with other commonly recommended interdental cleaning devices, such as interdental brushes. Further, certain limitations regarding the statistical analysis should be considered. Specifically, although, an adjustment (i.e., Knapp–Hartung standard error adjustment) to account for the small number of studies per comparison was performed, only two out of seven comparisons regarding the primary outcome parameters (i.e., interproximal plaque and interproximal bleeding) presented with more than three studies. Consequently, differentiation by a meta‐regression between manual and electric toothbrushes or between the different types of liquid‐based PDICT (i.e., oral irrigators with a jet stream of water at low velocity vs. oral irrigators emitting a microburst of high‐elocity air and liquid microdroplets) was not meaningful herein either. Similarly, no assumptions can be made on the possible efficacy of PDICT in other patient categories, such as periodontitis patients (Costa et al., 2020), orthodontic patients (Kossack & Jost‐Brinkmann, 2005; Sharma et al., 2008), or implant patients. Nevertheless, in regards to implant patients, where the use of PDICT seems relevant due to the often‐existing difficulty in proper access to the interproximal space, several recent studies presented results overall in favor of PDICT, for example, either for maintaining peri‐implant health (Salles et al., 2021) or in the treatment of peri‐implant mucositis (Bunk et al., 2020; Magnuson et al., 2013; Tütüncüoğlu et al., 2022). Thus, studies including comparisons with other relevant NPDICT (e.g., interdental brushes) and reporting on interproximal values, long‐term data, patient‐reported outcome measures, and other patient groups, are needed.

In conclusion, considering the limited number of original studies and/or comparisons, the following conclusions can be drawn regarding the use of PDICT in gingivitis patients:

− Liquid‐based PDICT significantly reduces interproximal inflammation compared with brushing alone; however, this seems not to be due to better plaque control.
− Liquid‐based and mechanical PDICT show a similar efficacy as flossing in terms of plaque and inflammation control.
− PDICT may achieve higher patient acceptance/compliance.

## AUTHOR CONTRIBUTIONS

**Pia Edlund**: Conceptualization; methodology; investigation; writing (original draft); visualization. **Kristina Bertl**: Conceptualization; methodology; formal analysis; investigation; data curation; writing (original draft); visualization. **Nikolaos Pandis**: Formal analysis; data curation; writing (review and editing); visualization. **Andreas Stavropoulos**: Conceptualization; methodology; validation; resources; writing (review and editing); supervision; project administration.

## CONFLICT OF INTEREST

The authors declare no conflict of interest.

## Supporting information

Supporting information.

Supporting information.

Supporting information.

Supporting information.

Supporting information.

Supporting information.

## Data Availability

Data are available from the authors upon reasonable request.
