# Supplementary material for: Efficacy of power‐driven interdental cleaning tools: A systematic review and meta‐analysis
Source: Clin Exp Dent Res. 2022 Dec 23;9(1):3–16. doi: 10.1002/cre2.691 (PMC9932241; doi:10.1002/cre2.691)
Supplement: Supplementary file 2 — Supporting information. [file CRE2-9-3-s003.docx]

**Appendix 2.** Details on the search term.

The following keywords were used with the filter function “humans”: ((mechanical interdental cleaning OR mechanical approximal cleaning OR mechanical interproximal cleaning OR mechanical interdental device* OR mechanical approximal device* OR mechanical interproximal device* OR mechanical approximal tool* OR mechanical interdental tool* OR mechanical interproximal tool* OR power driven interdental cleaning OR power driven approximal cleaning OR power driven interproximal cleaning OR power driven interdental device OR power driven approximal device OR power driven interproximal device OR power driven approximal tool* OR power driven interdental tool* OR power driven interproximal tool* OR electric interdental cleaning OR electric approximal cleaning OR electric interproximal cleaning OR electric interdental device* OR electric approximal device* OR electric interproximal device* OR electric approximal tool* OR electric interdental tool* OR electric interproximal tool* OR approximal cleaning device* OR interproximal cleaning device* OR interdental cleaning device* OR approximal cleaning aid* OR interproximal cleaning aid* OR interdental cleaning aid* OR oral irrigator OR oral irrigation OR oral irrigation jet OR water jet irrigator OR monojet oral irrigator OR dental water jet OR dental irrigator OR dental irrigation OR water pik OR waterpik OR perio pik OR periopik OR water jet OR water flosser OR air floss OR airfloss) AND (Periodontal disease OR periodontal disease[MeSH Terms] OR gingivitis OR gingivitis[MeSH Terms] OR plaque OR dental plaque[MeSH Terms] OR dental deposits[MeSH Terms] OR bleeding index OR plaque index OR dental plaque index[MeSH Terms]) NOT (endodontic OR root canal OR pulp)).
