# Supplementary material for: Efficacy of power‐driven interdental cleaning tools: A systematic review and meta‐analysis
Source: Clin Exp Dent Res. 2022 Dec 23;9(1):3–16. doi: 10.1002/cre2.691 (PMC9932241; doi:10.1002/cre2.691)
Supplement: Supplementary file 3 — Supporting information. [file CRE2-9-3-s004.docx]

**Appendix 3.** Reasons for exclusion of 13 full-texts.

| **Author (Year)** | **Reason for exclusion** |
| --- | --- |
| Aziz-Gandour (1986) | Patients with chronic periodontitis |
| Ciancio (1989) | No group without PDICT |
| Flemmig (1990) | Not randomized |
| Newman (1990) | Not randomized |
| Jackson (1991) | Not randomized |
| Watt (1993) | No group without PDICT |
| Chaves (1994) | Focus on chemical agents |
| Kossack (2005) | Patients undergoing orthodontic treatment |
| Sasikumar (2016) | Unclear methodology |
| Sharma (2012) | No group without PDICT |
| Sharma (2012) | No group without PDICT |
| Magnuson (2013) | Focus on implants |
| Goyal (2015) | No group without PDICT |

*PDICT, power-driven interdental cleaning tool.*

**References**

Aziz-Gandour, I. A., & Newman, H. N. (1986) The effects of a simplified oral hygiene regime plus supragingival irrigation with chlorhexidine or metronidazole on chronic inflammatory periodontal disease. *J Clin Periodontol* **13**, 228-236.

Chaves, E. S., Kornman, K. S., Manwell, M. A., Jones, A. A., Newbold, D. A., & Wood, R. C. (1994) Mechanism of irrigation effects on gingivitis. *J Periodontol* **65**, 1016-1021.

Ciancio, S. G., Mather, M. L., Zambon, J. J., & Reynolds, H. S. (1989) Effect of a chemotherapeutic agent delivered by an oral irrigation device on plaque, gingivitis, and subgingival microflora. *J Periodontol* **60**, 310-315.

Flemmig, T. F., Newman, M. G., Doherty, F. M., Grossman, E., Meckel, A. H., & Bakdash, M. B. (1990) Supragingival irrigation with 0.06% chlorhexidine in naturally occurring gingivitis. I. 6 month clinical observations. *J Periodontol* **61**, 112-117.

Goyal, C. R., Lyle, D. M., Qaqish, J. G., & Schuller, R. (2015) Efficacy of Two Interdental Cleaning Devices on Clinical Signs of Inflammation: A Four-Week Randomized Controlled Trial. *J Clin Dent* **26**, 55-60.

Jackson, C. L. (1991) Comparison between electric toothbrushing and manual toothbrushing, with and without oral irrigation, for oral hygiene of orthodontic patients. *Am J Orthod Dentofacial Orthop* **99**, 15-20.

Kossack, C., & Jost-Brinkmann, P. G. (2005) Plaque and gingivitis reduction in patients undergoing orthodontic treatment with fixed appliances-comparison of toothbrushes and interdental cleaning aids. A 6-month clinical single-blind trial. *J Orofac Orthop* **66**, 20-38.

Magnuson, B., Harsono, M., Stark, P. C., Lyle, D., Kugel, G., & Perry, R. (2013) Comparison of the effect of two interdental cleaning devices around implants on the reduction of bleeding: a 30-day randomized clinical trial. *Compend Contin Educ Dent* **34 Spec No 8**, 2-7.

Newman, M. G., Flemmig, T. F., Nachnani, S. et al. (1990) Irrigation with 0.06% chlorhexidine in naturally occurring gingivitis. II. 6 months microbiological observations. *J Periodontol* **61**, 427-433.

Sasikumar, P. K., Shanmugam, S., Devi, S. S., & Kirthika, M. (2016) Comparative Evaluation of Oral Irrigator and Dental Floss as an adjunct to Tooth Brushing on Reduction of Plaque and Gingivitis- A Randomized, Single Blind Clinical Study of Rural Patients. *Journal of Applied Dental and Medical Sciences* **2**, 51-61.

Sharma, N. C., Lyle, D. M., Qaqish, J. G., & Schuller, R. (2012a) Comparison of two power interdental cleaning devices on plaque removal. *J Clin Dent* **23**, 17-21.

Sharma, N. C., Lyle, D. M., Qaqish, J. G., & Schuller, R. (2012b) Comparison of two power interdental cleaning devices on the reduction of gingivitis. *J Clin Dent* **23**, 22-26.

Watt, D. L., Rosenfelder, C., & Sutton, C. D. (1993) The effect of oral irrigation with a magnetic water treatment device on plaque and calculus. *J Clin Periodontol* **20**, 314-317.
