# Supplementary material for: Efficacy of power‐driven interdental cleaning tools: A systematic review and meta‐analysis
Source: Clin Exp Dent Res. 2022 Dec 23;9(1):3–16. doi: 10.1002/cre2.691 (PMC9932241; doi:10.1002/cre2.691)
Supplement: Supplementary file 4 — Supporting information. [file CRE2-9-3-s001.docx]

**Appendix 4.** Risk of bias (RoB) assessment of the randomized controlled clinical trials (Cochrane Collaboration’s RoB 2.0 tool); RoB assessment of a) the individual studies, and b) overall.

**
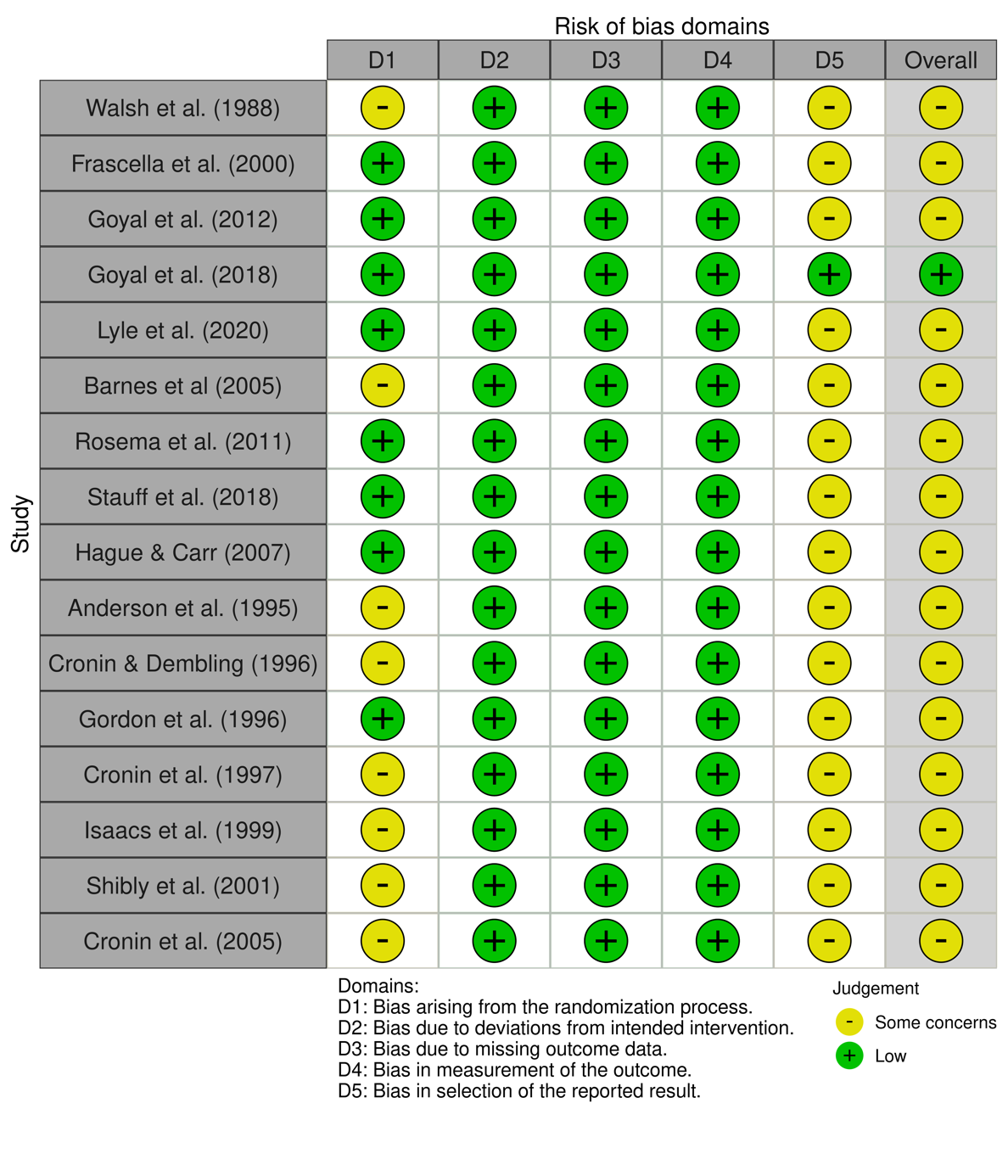
**

*
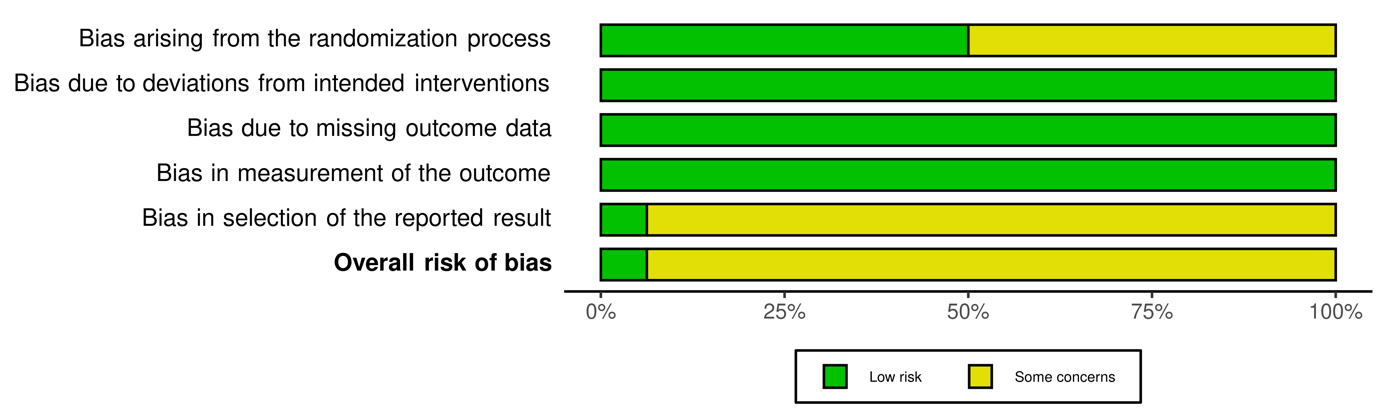
*
