# Supplementary material for: Efficacy of power‐driven interdental cleaning tools: A systematic review and meta‐analysis
Source: Clin Exp Dent Res. 2022 Dec 23;9(1):3–16. doi: 10.1002/cre2.691 (PMC9932241; doi:10.1002/cre2.691)
Supplement: Supplementary file 5 — Supporting information. [file CRE2-9-3-s005.docx]

**Appendix 5.** Synthesis of the secondary outcome variables.

Interproximal gingival index and full mouth plaque, bleeding, and gingival indices were defined as secondary outcome variables. Meta-analyses were possible for comparison 1 (interproximal gingival indices, full mouth plaque, bleeding, and gingival indices), comparison 2 (full mouth plaque indices), and comparison 4 (interproximal gingival indices, full mouth plaque, bleeding, and gingival indices).

*Comparison 1. Brushing vs. brushing + liquid-based PDICT*

Two studies (Goyal et al. 2018; Lyle et al. 2020) assessed interproximal gingival indices (Appendix Figure 1). Both studies presented a positive effect but had a significant heterogeneity among each other (I^2^ 69.4%, p = 0.07). Meta-analysis failed to show a significant positive effect favoring the adjunct use of a liquid-based PDICT [SMD -0.93 (95% CI: -4.96, 3.11); p = 0.21].

Four studies (Frascella et al. 2000; Goyal et al. 2012; Goyal et al. 2018; Lyle et al. 2020) assessed full mouth plaque (Appendix Figure 2). All studies presented a positive effect but with a significant heterogeneity among each other (I^2^ 86.3%, p < 0.01). Meta-analysis showed no significant difference between the two interventions [SMD -0.58 (95% CI: -1.65, 0.48); p = 0.18].

Four studies (Frascella et al. 2000; Goyal et al. 2012; Goyal et al. 2018; Lyle et al. 2020) assessed full mouth bleeding (Appendix Figure 3). All studies presented a positive effect but with a significant heterogeneity among them (I^2^ 94.9%, p < 0.01). Meta-analysis showed a significant positive effect favoring the adjunct use of a liquid-based PDICT [SMD -2.30 (95% CI: -4.51, -0.10); p = 0.04].

Finally, 4 studies (Frascella et al. 2000; Goyal et al. 2012; Goyal et al. 2018; Lyle et al. 2020) assessed full mouth gingival indices (Appendix Figure 4). All studies reported a positive effect but with a significant heterogeneity among each other (I^2^ 78.5%, p < 0.01). Meta-analysis showed a tendency for a positive effect favoring the adjunct use of a liquid-based PDICT [SMD -0.72 (95% CI: -1.58, 0.13) p = 0.07].

*Comparison 2. Brushing + flossing vs. brushing + liquid-based PDICT*

Two studies (Barnes et al. 2005; Rosema et al. 2011) assessed full mouth plaque (Appendix Figure 2). Both studies showed a tendency to favour the control group without significant heterogeneity among each other (I^2^ 0%, p = 0.35). Meta-analysis showed no significant difference between the two interventions [SMD 0.26 (95% CI: -2.01, 2.52); p = 0.39].

*Comparison 4. Brushing + flossing vs. brushing + mechanical PDICT.*

Five studies (Gordon et al. 1996; Cronin et al. 1997; Shibly et al. 2001; Cronin et al. 2005; Hague & Carr 2007) contributed with 6 comparisons on interproximal gingival indices (Appendix Figure 1). The studies reported controversial results but presented no significant heterogeneity (I^2^ 0%, p = 0.65). Meta-analysis showed no significant difference between the two interventions [SMD 0.04 (95% CI: -0.22, 0.29); p = 0.73].

Four studies (Anderson et al. 1995; Cronin et al. 1997; Shibly et al. 2001; Cronin et al. 2005) contributed with 5 comparisons on full mouth plaque (Appendix Figure 2). The studies reported controversial results but presented no significant heterogeneity (I^2^ 23.5%, p = 0.31). Meta-analysis showed no significant difference between the two interventions [SMD 0.15 (95% CI: -0.24, 0.55) p = 0.34].

Two studies (Cronin et al. 1997; Cronin et al. 2005) contributed with 3 comparisons on full mouth bleeding (Appendix Figure 3). The studies reported controversial results but presented no significant heterogeneity (I^2^ 18.0%, p = 0.31). Meta-analysis showed no significant difference between the two interventions [SMD 0.00 (95% CI: -0.87, 0.86) p = 0.99].

Three studies (Cronin et al. 1997; Shibly et al. 2001; Cronin et al. 2005) contributed with 4 comparisons on full mouth gingival indices (Appendix Figure 4). The studies reported controversial results but presented no significant heterogeneity (I^2^ 21.8 %, p = 0.29). Meta-analysis showed no significant difference between the two interventions [SMD 0.10 (CI: -0.43, 0.64) p = 0.58].


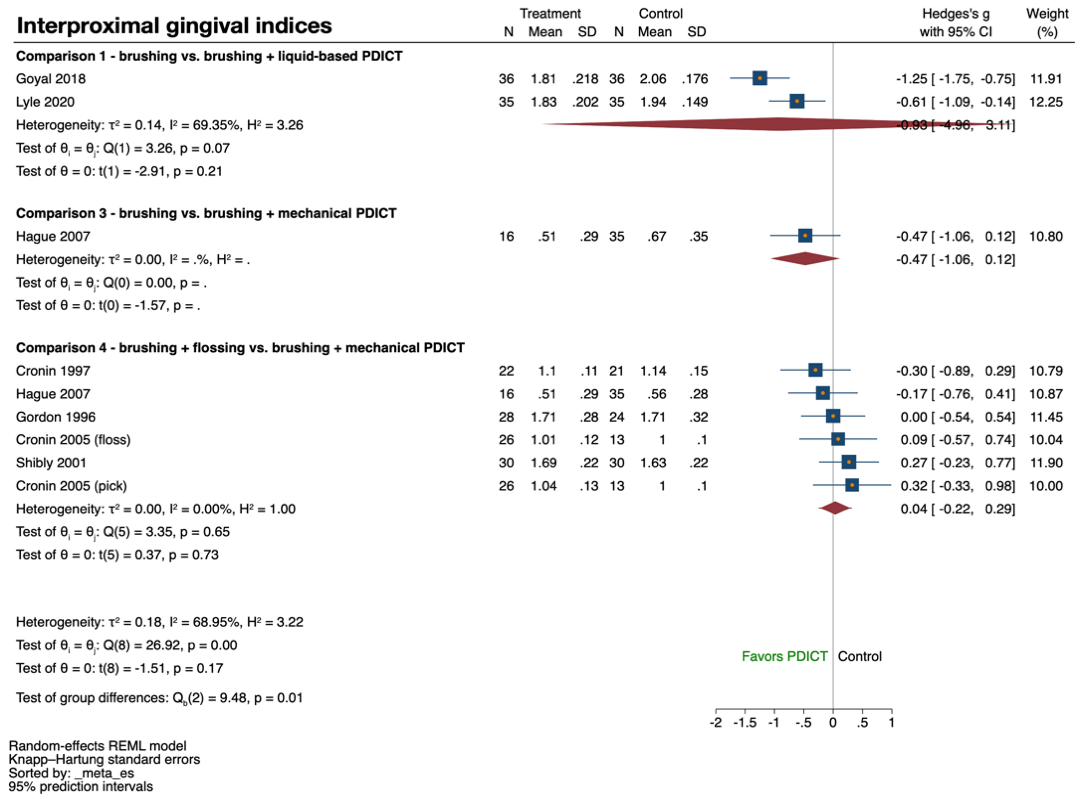


**Appendix Figure 1.** Forest plot for interproximal gingival indices.

**
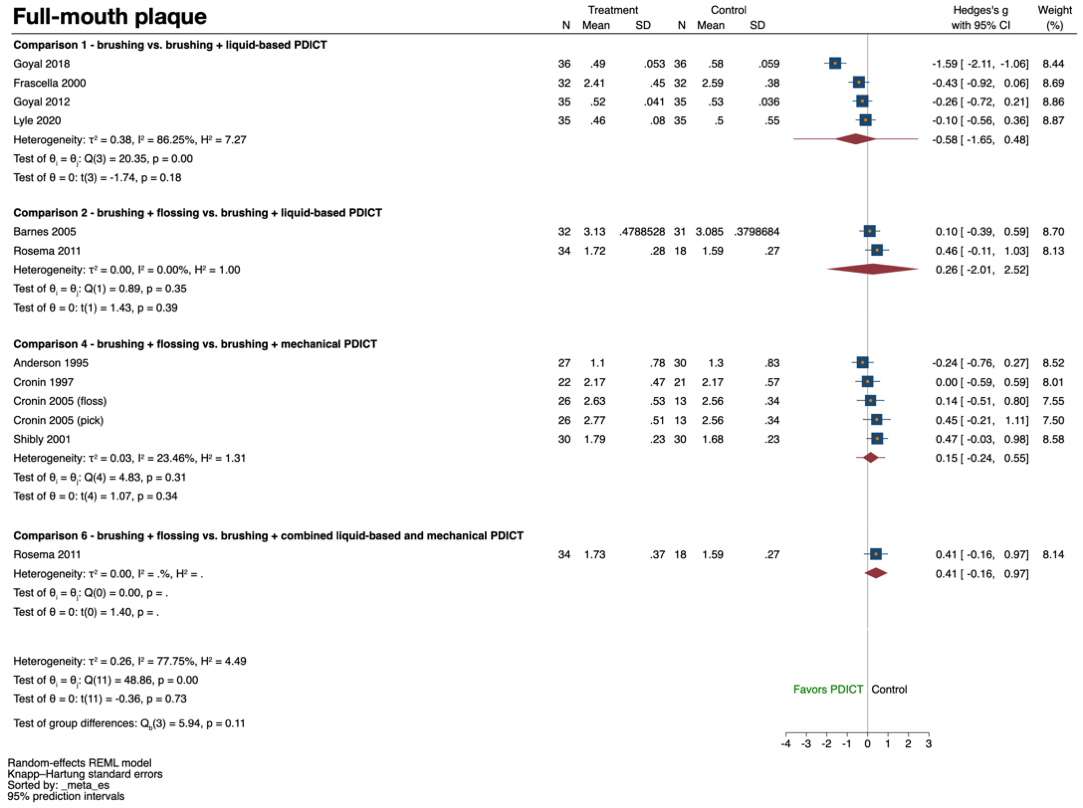
**

**Appendix Figure 2.** Forest plot for full mouth plaque.


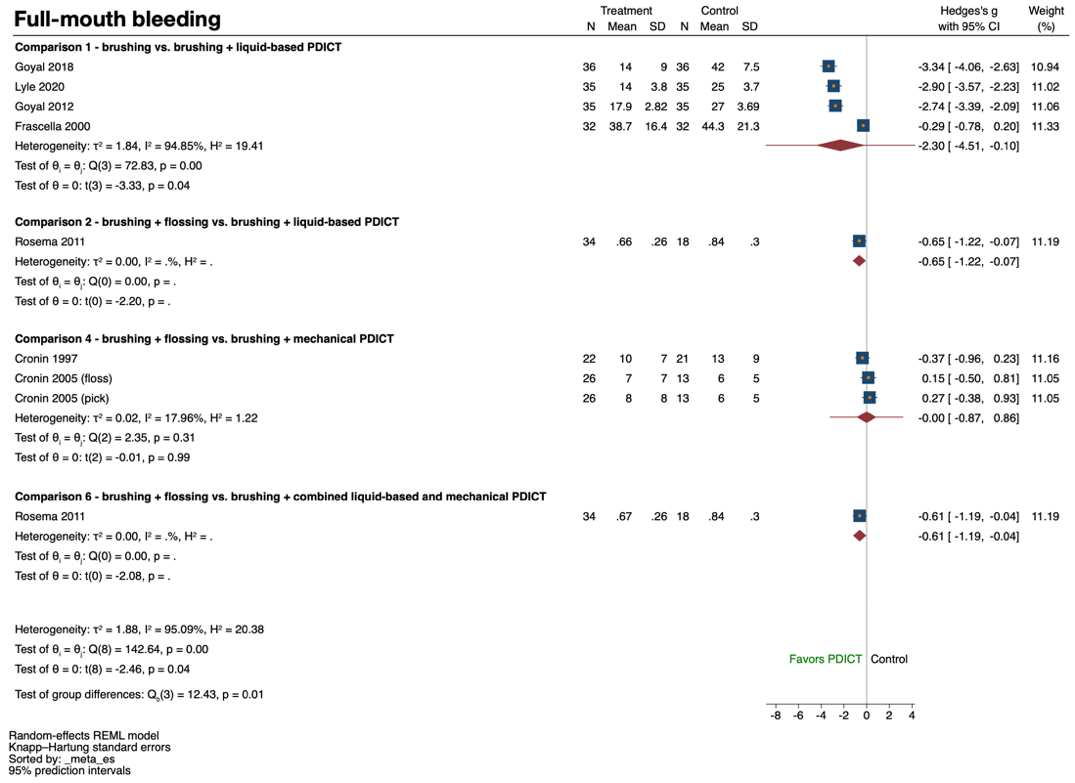


**Appendix Figure 3.** Forest plot for full mouth bleeding.


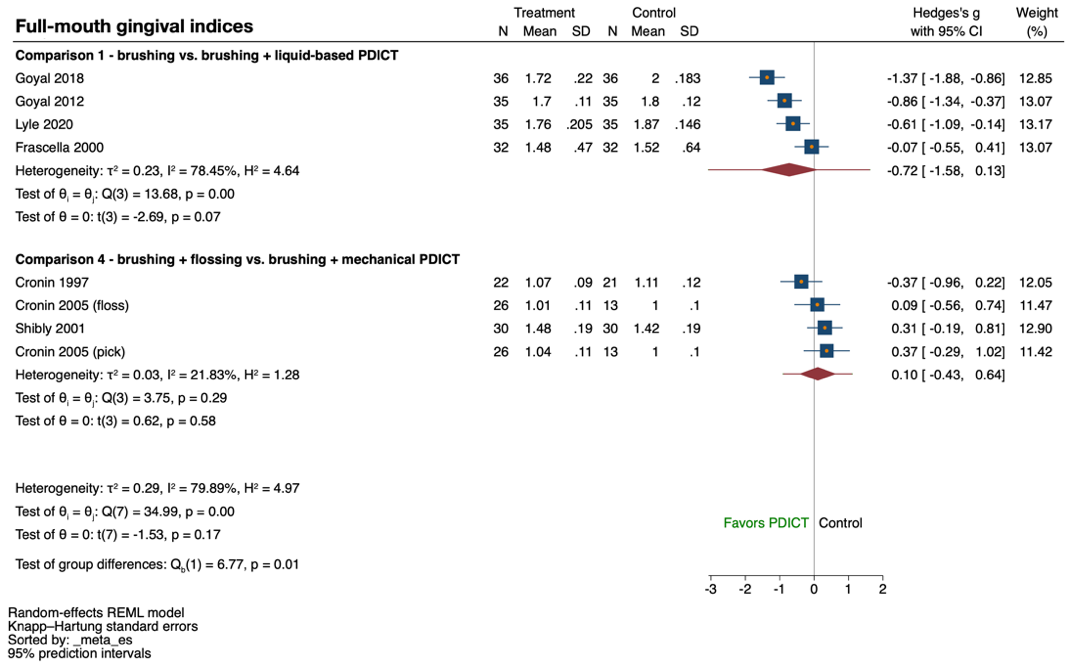


**Appendix Figure 4.** Forest plot for full mouth gingival indices.

**References**

Anderson, N. A., Barnes, C. M., Russell, C. M., & Winchester, K. R. (1995) A clinical comparison of the efficacy of an electromechanical flossing device or manual flossing in affecting interproximal gingival bleeding and plaque accumulation. *J Clin Dent* **6**, 105-107.

Barnes, C. M., Russell, C. M., Reinhardt, R. A., Payne, J. B., & Lyle, D. M. (2005) Comparison of irrigation to floss as an adjunct to tooth brushing: effect on bleeding, gingivitis, and supragingival plaque. *J Clin Dent* **16**, 71-77.

Cronin, M., Dembling, W., & Warren, P. (1997) The safety and efficacy of gingival massage with an electric interdental cleaning device. *J Clin Dent* **8**, 130-133.

Cronin, M. J., Dembling, W. Z., Cugini, M., Thompson, M. C., & Warren, P. R. (2005) A 30-day clinical comparison of a novel interdental cleaning device and dental floss in the reduction of plaque and gingivitis. *J Clin Dent* **16**, 33-37.

Frascella, J. A., Fernández, P., Gilbert, R. D., & Cugini, M. (2000) A randomized, clinical evaluation of the safety and efficacy of a novel oral irrigator. *Am J Dent* **13**, 55-58.

Gordon, J. M., Frascella, J. A., & Reardon, R. C. (1996) A clinical study of the safety and efficacy of a novel electric interdental cleaning device. *J Clin Dent* **7**, 70-73.

Goyal, C. R., Lyle, D. M., Qaqish, J. G., & Schuller, R. (2012) The addition of a water flosser to power tooth brushing: effect on bleeding, gingivitis, and plaque. *J Clin Dent* **23**, 57-63.

Goyal, C. R., Qaqish, J. G., Schuller, R., & Lyle, D. M. (2018) Evaluation of the Addition of a Water Flosser to Manual Brushing on Gingival Health. *J Clin Dent* **29**, 81-86.

Hague, A. L., & Carr, M. P. (2007) Efficacy of an automated flossing device in different regions of the mouth. *J Periodontol* **78**, 1529-1537.

Lyle, D. M., Qaqish, J. G., Goyal, C. R., & Schuller, R. (2020) Efficacy of the Use of a Water Flosser in Addition to an Electric Toothbrush on Clinical Signs of Inflammation: 4-Week Randomized Controlled Trial. *Compend Contin Educ Dent* **41**, 170-177.

Rosema, N. A., Hennequin-Hoenderdos, N. L., Berchier, C. E., Slot, D. E., Lyle, D. M., & van der Weijden, G. A. (2011) The effect of different interdental cleaning devices on gingival bleeding. *J Int Acad Periodontol* **13**, 2-10.

Shibly, O., Ciancio, S. G., Shostad, S., Mather, M., & Boardman, T. J. (2001) Clinical evaluation of an automatic flossing device vs. manual flossing. *J Clin Dent* **12**, 63-66.
