# Supplementary material for: Efficacy of power‐driven interdental cleaning tools: A systematic review and meta‐analysis
Source: Clin Exp Dent Res. 2022 Dec 23;9(1):3–16. doi: 10.1002/cre2.691 (PMC9932241; doi:10.1002/cre2.691)
Supplement: Supplementary file 6 — Supporting information. [file CRE2-9-3-s002.docx]

**Appendix 6.** Quality of Evidence (GRADE) / Summary of Findings Tables.

1. **Comparison 1 - brushing vs. brushing + liquid-based PDICT**

| **Summary of findings:** | | | | | | |
| --- | --- | --- | --- | --- | --- | --- |
| **Brushing + liquid-based PDICT compared to brushing in gingivitis patients** | | | | | | |
| **Patient or population:** gingivitis patients  **Setting:**  **Intervention:** brushing + liquid-based PDICT  **Comparison:** brushing | | | | | | |
| Outcomes | **Anticipated absolute effects^*^** (95% CI) | | Relative effect (95% CI) | № of participants (studies) | Certainty of the evidence (GRADE) | Comments |
|  | **Risk with brushing** | **Risk with brushing + liquid-based PDICT** |  |  |  |  |
| Interproximal plaque follow-up: 30 days | - | SMD **0.8 SD lower** (2.92 lower to 1.31 higher) | - | 212 (3 RCTs) | ⨁◯◯◯ Very low^a,b,c^ |  |
| Interproximal bleeding follow-up: 30 days | - | SMD **3.16 SD lower** (4.73 lower to 1.6 lower) | - | 142 (2 RCTs) | ⨁⨁⨁◯ Moderate^d^ |  |
| ***The risk in the intervention group** (and its 95% confidence interval) is based on the assumed risk in the comparison group and the **relative effect** of the intervention (and its 95% CI).  **CI:** confidence interval; **SMD:** standardised mean difference | | | | | | |
| **GRADE Working Group grades of evidence** **High certainty:** we are very confident that the true effect lies close to that of the estimate of the effect. **Moderate certainty:** we are moderately confident in the effect estimate: the true effect is likely to be close to the estimate of the effect, but there is a possibility that it is substantially different. **Low certainty:** our confidence in the effect estimate is limited: the true effect may be substantially different from the estimate of the effect. **Very low certainty:** we have very little confidence in the effect estimate: the true effect is likely to be substantially different from the estimate of effect. | | | | | | |

#### Explanations

a. 2 out of 3 studies presenting with some concerns

b. high heterogeneity

c. due to wide 95% CI

d. 1 out of 2 studies presenting with some concerns

1. **Comparison 2 - brushing + flossing vs. brushing + liquid-based PDICT**

| **Summary of findings:** | | | | | | |
| --- | --- | --- | --- | --- | --- | --- |
| **Brushing + liquid-based PDICT compared to brushing + flossing in gingivitis patients** | | | | | | |
| **Patient or population:** gingivitis patients  **Setting:**  **Intervention:** brushing + liquid-based PDICT  **Comparison:** brushing + flossing | | | | | | |
| Outcomes | **Anticipated absolute effects^*^** (95% CI) | | Relative effect (95% CI) | № of participants (studies) | Certainty of the evidence (GRADE) | Comments |
|  | **Risk with brushing + flossing** | **Risk with brushing + liquid-based PDICT** |  |  |  |  |
| Interproximal bleeding follow-up: 30 days | - | SMD **0.36 SD lower** (1.17 lower to 0.45 higher) | - | 123 (2 RCTs) | ⨁⨁◯◯ Low^a,b^ |  |
| ***The risk in the intervention group** (and its 95% confidence interval) is based on the assumed risk in the comparison group and the **relative effect** of the intervention (and its 95% CI).  **CI:** confidence interval; **SMD:** standardised mean difference | | | | | | |
| **GRADE Working Group grades of evidence** **High certainty:** we are very confident that the true effect lies close to that of the estimate of the effect. **Moderate certainty:** we are moderately confident in the effect estimate: the true effect is likely to be close to the estimate of the effect, but there is a possibility that it is substantially different. **Low certainty:** our confidence in the effect estimate is limited: the true effect may be substantially different from the estimate of the effect. **Very low certainty:** we have very little confidence in the effect estimate: the true effect is likely to be substantially different from the estimate of effect. | | | | | | |

#### Explanations

a. all studies presenting with some concerns

b. due to wide 95% CI

1. **Comparison 4 - brushing + flossing vs. brushing + mechanical PDICT**

| **Summary of findings:** | | | | | | |
| --- | --- | --- | --- | --- | --- | --- |
| **Brushing + mechanical PDICT compared to brushing + flossing in gingivitis patients** | | | | | | |
| **Patient or population:** gingivitis patients  **Setting:**  **Intervention:** brushing + mechanical PDICT  **Comparison:** brushing + flossing | | | | | | |
| Outcomes | **Anticipated absolute effects^*^** (95% CI) | | Relative effect (95% CI) | № of participants (studies) | Certainty of the evidence (GRADE) | Comments |
|  | **Risk with brushing + flossing** | **Risk with brushing + mechanical PDICT** |  |  |  |  |
| Interproximal plaque follow-up: 30 days | - | SMD **0.08 SD higher** (0.43 lower to 0.6 higher) | - | 284 (5 RCTs) | ⨁⨁◯◯ Low^a,b^ |  |
| Interproximal bleeding follow-up: 30 days | - | SMD **0 SD**  (0.29 lower to 0.28 higher) | - | 290 (5 RCTs) | ⨁⨁◯◯ Low^a,c^ |  |
| ***The risk in the intervention group** (and its 95% confidence interval) is based on the assumed risk in the comparison group and the **relative effect** of the intervention (and its 95% CI).  **CI:** confidence interval; **SMD:** standardised mean difference | | | | | | |
| **GRADE Working Group grades of evidence** **High certainty:** we are very confident that the true effect lies close to that of the estimate of the effect. **Moderate certainty:** we are moderately confident in the effect estimate: the true effect is likely to be close to the estimate of the effect, but there is a possibility that it is substantially different. **Low certainty:** our confidence in the effect estimate is limited: the true effect may be substantially different from the estimate of the effect. **Very low certainty:** we have very little confidence in the effect estimate: the true effect is likely to be substantially different from the estimate of effect. | | | | | | |

#### Explanations

a. all studies presenting with some concerns

b. due to high heterogeneity

c. due to unclear effect (95% CI)
